# Vascular calcification is not associated with increased ambulatory central aortic systolic pressure in prevalent dialysis patients

**DOI:** 10.5830/CVJA-2013-081

**Published:** 2014-02

**Authors:** Robert J Freercks, Charles R Swanepoel, Kristy L Turest-Swartz, Brian L Rayner, Henri RO Carrara, Sulaiman EI Moosa, Anthony S Lachman

**Affiliations:** Renal Unit, Groote Schuur Hospital, University of Cape Town, South Africa; Renal Unit, Groote Schuur Hospital, University of Cape Town, South Africa; Renal Unit, Groote Schuur Hospital, University of Cape Town, South Africa; Renal Unit, Groote Schuur Hospital, University of Cape Town, South Africa; School of Public Health and Family Medicine, University of Cape Town, South Africa; Radiologist, 2-Military Hospital, Cape Town; Cardiologist, 2-Military Hospital, Cape Town

**Keywords:** vascular calcification, central blood pressure, dialysis, ambulatory blood pressure monitoring

## Abstract

**Introduction:**

Central aortic systolic pressure (CASP) strongly predicts cardiovascular outcomes. We undertook to measure ambulatory CASP in 74 prevalent dialysis patients using the BPro (HealthStats, Singapore) device. We also determined whether coronary or abdominal aortic calcification was associated with changes in CASP and whether interdialytic CASP predicted ambulatory measurement.

**Methods:**

All patients underwent computed tomography for coronary calcium score, lateral abdominal radiography for aortic calcium score, echocardiography for left ventricular mass index and ambulatory blood pressure measurement using BPro calibrated to brachial blood pressure. HealthStats was able to convert standard BPro SOFT® data into ambulatory CASP.

**Results:**

Ambulatory CASP was not different in those without and with coronary (137.6 vs 141.8 mmHg, respectively, *p* = 0.6) or aortic (136.6 vs 145.6 mmHg, respectively, *p* = 0.2) calcification. Furthermore, when expressed as a percentage of brachial systolic blood pressure to control for peripheral blood pressure, any difference in CASP was abolished: CASP: brachial systolic blood pressure ratio = 0.9 across all categories regardless of the presence of coronary or aortic calcification (*p* = 0.2 and 0.4, respectively). Supporting this finding, left ventricular mass index was also not different in those with or without vascular calcification (*p* = 0.7 and 0.8 for coronary and aortic calcification). Inter-dialytic office blood pressure and CASP correlated excellently with ambulatory measurements (*r* = 0.9 for both).

**Conclusion:**

Vascular calcification was not associated with changes in ambulatory central aortic systolic pressure in this cohort of prevalent dialysis patients. Inter-dialytic blood pressure and CASP correlated very well with ambulatory measurement.

## Abstract

Vascular calcification (VC) is a novel vascular risk factor strongly associated with mortality in dialysis patients.^1^,^2^ Although various explanations exist for this association, one mechanism is through alterations in pulse-wave velocity (PWV). Vascular calcification is associated with increased aortic PWV,^3^ which in turn is associated with raised central aortic systolic pressure (CASP) and reduced coronary perfusion.^4^,^5^ As a result, brachial pressure may significantly under- or over-estimate central pressure.^6^

Not surprisingly therefore, central blood pressure parameters have been shown to predict hard cardiovascular endpoints (including mortality) better than concomitant brachial measurements.^7^-^10^ Whether vascular calcification is directly linked to central pressures is, however, unknown since there are many determinants of aortic stiffening other than calcification. Furthermore, a primarily damaged and stiff aorta may be the target for secondary deposition of calcium.^11^

CASP can be calculated using applanation tonometry-derived peripheral pulse waveforms and associated software.^12^ This avoids the obvious disadvantages of invasive central pressure determination. The major disadvantage of standard techniques, however, is the one-dimensional static measurement that is obtained, with no information on ambulatory values or nocturnal dipping status.

Loss of normal nocturnal systolic blood pressure dipping is prevalent in chronic kidney disease (CKD) and likely contributes to cardiovascular disease.^13^ Dipping, which can only be assessed using ambulatory monitoring techniques, correlates better with left ventricular mass index (LVMI) in end-stage renal disease than office-based blood pressure measurement.^14^,^15^

There have been calls for the routine use of ambulatory blood pressure monitoring (ABPM) in clinical studies of CKD^13^,^16^ and indeed, for investigations into the utility of ambulatory CASP in clinical practice.^17^,^18^ Combining both ambulatory and central pressure measurements is an attractive strategy, but until recently has not been technically possible.

A non-invasive wrist watch-like device, BPro with A-Pulse CASP software (HealthStats, Singapore) was recently approved by the US Food and Drug Administration (FDA: K072593) for the measurement of CASP as well as ambulatory blood pressure. It is a small, wrist watch-like, cuffless monitor which obtains radial pressure waveforms by applanation tonometry. BPro has the ability to measure ambulatory CASP and although not yet commercially available, the manufacturer is able to convert data into ambulatory CASP using the same software.

As part of a recently published study on vascular calcification,^19^ we sought to prospectively evaluate whether the presence of vascular calcification had any relationship with ambulatory CASP in our young CKD-5D cohort using the BPro® radial pulse-wave acquisition device. We also sought to determine the utility of inter-dialytic office brachial and central blood pressure measurements in predicting ambulatory parameters.

## Methods

The study was approved by the Research Ethics Committee of the University of Cape Town, South Africa. The full methodology has been published elsewhere,^19^ but briefly, cases were selected if they were on maintenance dialysis of three months or longer duration and were able to sign informed consent. Seventy-five prevalent dialysis patients 18 years or older were enrolled from Groote Schuur Hospital, Cape Town.

Patients were excluded if they were pregnant or planning a pregnancy, had sustained arrhythmias or prior coronary stenting or bypass. One patient was excluded due to loss to follow up so the final case sample was 74 participants. Clinical and demographic data were collected and ethnicity was self-reported.

Ambulatory and office blood pressure monitoring: the BPro® radial pulse wave acquisition device and A-pulse CASP® software (HealthStats, Singapore) system uses an N-point moving-average method to non-invasively derive CASP from the radial arterial pressure waveform. It has been validated against a generalised transfer function method using CAFE study data as well as central aortic pressures recorded *in vivo* at the aortic root, using a Millar’s SPC–454D tonometer (Millar’s instruments, Texas USA).^20^ The device also recently compared favourably to the widely used non-invasive SphygmoCor system (AtCor Medical, Sydney, New South Wales, Australia), with good agreement compared to invasively determined CASP.^17^

For blood pressure determination, the BPro™ has been validated against the Association for the Advancement of Medical Instrumentation and European Society of Hypertension (ESH) protocols and passed both validations.^21^ The BPro™ records pressure wave forms calibrated to the brachial blood pressure and samples up to 96 × 10-second blocks of time over 24 hours. This provides a 24-hour profile and summary of an individual’s systolic, diastolic and mean arterial pressures via the use of BPro SOFT® software.

Practically, the device was applied on the non-dominant arm or that which did not contain an AVF on the inter-dialytic day for haemodialysis patients or at a routine visit for prevalent dialysis patients. The device was then calibrated to office blood pressure – brachial blood pressure obtained via use of the MC3000 oscillometric device (HealthStats) according to the recommended ESH protocol.^22^ The manufacturer was able to convert the ABPM data into ambulatory CASP readings since the data are acquired in the same way for both.

Cardiac CT and coronary calcium score: images were acquired using the Philips Brilliance 64-slice MDCT scanner. A standard protocol was used as follows: tube voltage, 120 kV; tube current, 55 mAs; detector collimation, 40 × 0.625 mm; gantry rotation, 400 ms. CT data were transferred to the Philips Extended Brilliance Workstation Version 4.0.2.145 for analysis and coronary calcium score was calculated with the Agatston algorithm.^23^ All scans were evaluated by a single experienced radiologist (SM) and the intra-reader variability was tested and was below 10%.

Abdominal X-ray and abdominal aortic calcium score: a standard technique of exposing the lateral lumbar spine in the standing position (with 100-cm film distance, 94 KVP, and 33–200 mAs) was used. Calcific deposits in the abdominal aorta were scored as described by Kaupilla,^24^ by a single experienced clinician (RF) blinded to clinical data and coronary calcium score.

Echocardiography: assessment of the left ventricular mass was done via use of M-mode echocardiography and this was calculated using the Penn convention.^25^ Left ventricular hypertrophy was defined as > 125 g/m^2^ in males and > 110 g/m^2^ in females as per ESH guidelines.^26^ All scans were obtained and evaluated by a single experienced cardiologist (AL).

## Statistical analysis

Normality was determined with the Shapiro–Wilk test. Continuous variables are expressed as mean ± SD or median and inter-quartile range (IQR) and compared with the two-tailed independent Student’s *t*-test and Mann–Whitney test as appropriate. Dichotomous data are presented as percentages and compared with chi-square tests. All analyses were conducted using Stata 12.0 statistical software (College Station, TX, USA).

## Results

Table 1 shows the baseline characteristics of all patients. Overall, only 27 patients (38.6%) in the cohort had a coronary calcium score ≥ 1, and 26 (35.6%) had an abdominal aortic calcium score ≥ 1. The median coronary calcium score in those with coronary calcification was 141 (IQR = 55–619) and in those with abdominal aortic calcification, the median abdominal aortic calcium score was 6 (IQR = 1–10). Table 2 shows the baseline characteristics for all subjects with and without coronary and/or abdominal aortic calcification.

**Table 1.** Baseline characteristics of patients (*n* = 74 unless otherwise indicated)

*Characteristic*	*Value*	*Range (SD/IQR)*
Age, mean (years)	41.8	10.5
Women (%)	56.8	
Months on dialysis, median	32.0	43.6
Diabetes (%)	13.5	
Tobacco use (%)	41.9	
History of cardiovascular disease (%)	4.0	
Office systolic BP (mmHg)	146.8	28.0
Office diastolic BP (mmHg)	95.2	17.6
ABPM systolic BP^a^ (mmHg)	147.4	33.1
ABPM diastolic BP^a^ (mmHg)	97.6	21.7
ABPM peripheral pulse pressure^a^ (mmHg)	49.8	15.4
ABPM central aortic systolic pressure^a^ (mmHg)	139.2	31.3
ABPM dipping status^a^ (%)	5.3	5.5
LVMI (g/m^2^)	180.4	97.4
(g/m2) 180.4 97.4 LVH		
By ECHO	86.4	
By ECG	70.3	
Number of antihypertensives used, mean	2.3	1.4

SD, standard deviation; IQR, interquartile range; ABPM, ambulatory blood pressure monitoring; BP, blood pressure; LVMI, left ventricular mass index; LVH, left ventricular hypertrophy. ^a^*n* = 72.

**Table 2.** Baseline characteristics by presence of vascular calcification

*Variable*	*Coronary calcification*					*Abdominal aortic calcification*				
	*n*	*CAC^–^*	*n*	*CAC^++^*	p*-value*	*n*	*AAC^–^*	*n*	*AAC^++^*	p*-value*
Age (median)	43	38.3	27	46.0	< 0.01	47	39.3	26	46.0	< 0.01
Age (median)	43	1.1	27	0.5	0.1	47	1.0	26	0.4	0.1
Tobacco use (ever) (%)	43	37.2	27	51.9	0.2	47	34.0	26	57.7	0.1
Prior cardiovascular events (%)	43	2.3	27	7.4	0.3	47	4.3	26	3.9	0.9
Presence of diabetes (%)	43	7.0	27	25.9	< 0.05	47	4.3	26	30.8	< 0.01
Office systolic BP (mmHg)	43	145.5	26	149.0	0.6	46	144.1	25	152.8	0.2
Office diastolic BP (mmHg)	43	95.4	26	94.8	0.9	46	94.5	25	96.5	0.7
Office central aortic systolic pressure (mmHg)	43	132.8	26	134.9	0.7	46	131.4	25	138.2	0.3
ABPM systolic BP (mmHg)	43	145.8	26	150.1	0.6	46	144.4	25	154.2	0.2
ABPM diastolic BP, mmHg	43	97.7	26	97.3	0.9	46	96.7	25	99.6	0.5
ABPM peripheral pulse pressure (mmHg)	43	48.0	26	52.8	0.2	46	39.7	25	46.0	0.1
ABPM central aortic systolic pressure (mmHg)	43	137.6	26	141.8	0.6	46	136.3	25	145.6	0.2
ABPM central aortic systolic/systolic pressure ratio	43	0.9	26	0.9	0.2	46	0.9	25	0.9	0.4
Nocturnal systolic dipping (%)	37	6.2	25	4.0	0.1	40	5.8	24	4.5	0.3
Left ventricular mass index (g/m^2^)	43	179.7	26	187.1	0.7	45	188.0	25	198.0	0.8
LVH on echocardiography (%)	43	81.4	27	92.6	0.2	47	85.1	26	88.5	0.7

CAC–, coronary artery calcium score = 0; CAC^++^, coronary artery calcium score = ≥1; AAC^-^, abdominal aortic calcium score = 0; AAC^++^, abdominal aortic calcium score = ≥1; m:f = male:female; ABPM, ambulatory blood pressure monitoring; BP, blood pressure.

Both coronary and aortic calcium presence failed to show any association with CASP (*p* = 0.2 and 0.4, respectively). There was no difference when the ratio was compared in those with the highest versus lowest quartiles of coronary and aortic calcification (*p* = 0.2). Furthermore, there was no difference in the absolute difference between ambulatory systolic blood pressure and CASP values between those with and without coronary calcification (difference = 8.27 and 8.16, respectively, *p* = 0.8).

**Fig. 1.**
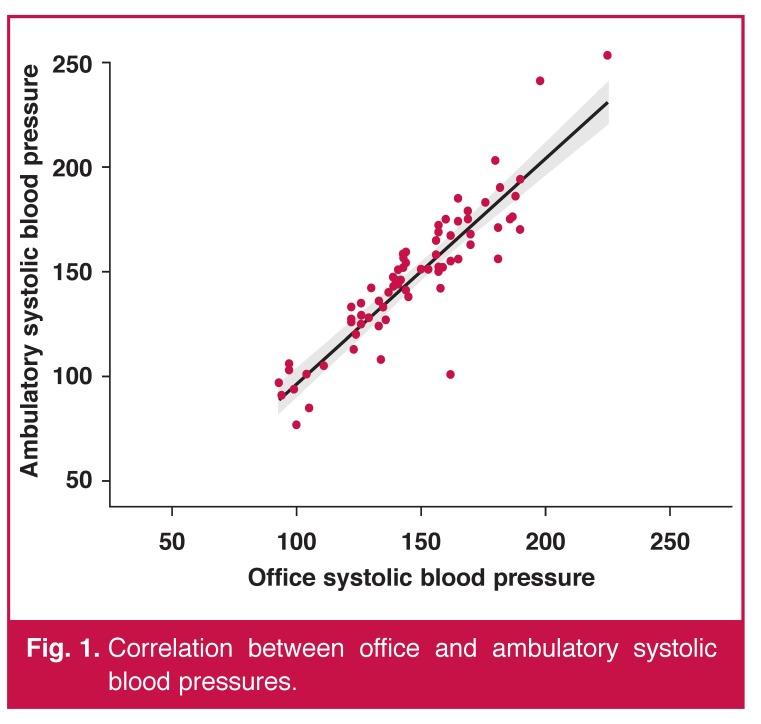
Correlation between office and ambulatory systolic blood pressures.

Fig. 1. shows the correlation of office with ambulatory systolic blood pressure. Office systolic blood pressure and CASP correlated well with their ambulatory measurement (both *r* = 0.90).

## Discussion

This was an observational study of 75 consecutive patients undergoing dialysis in a South African public sector unit. The cohort was young with a low level of co-morbidity due to stringent criteria for the selection of dialysis patients.

A key finding in this study was that both coronary and abdominal aortic calcification was not associated with a higher CASP relative to the brachial systolic blood pressure. This ratio was used to control for systolic blood pressure, which would otherwise make comparison between groups difficult. Since the study had an 80% power to detect a difference of > 3% in CASP, it was unlikely that there would be a clinically meaningful difference between CASP values with and without calcification.

The reasons for these findings are unclear but may be that vascular calcification is not directly responsible for aortic stiffening and the association of calcification with PWV is not causative. There are many other factors such as elastin fragmentation, endothelial dysfunction and advanced glycation that affect aortic stiffness other than calcification.^11^

Alternatively, since vascular micro-calcifications may be present in uraemic subjects without radiologically visible calcium,^27^ it is possible that vascular stiffening occurs earlier on and obscures any differences in CASP.

Unfortunately, we were unable to measure PWV. As left ventricular mass index is strongly determined by CASP,^8^,^28^ the lack of association with vascular calcification supports our controversial findings.

Non-dipping was particularly prevalent, as in other studies of CKD,^29^ and although it has been associated with vascular calcification,^30^ it was not different in those with and without vascular calcification in this cohort. However, the very poor dipping status overall may have obscured any clinically meaningful difference between the two groups.

Both inter-dialytic office blood pressure and CASP correlated well with ambulatory blood pressure measurements. This has important implications since the FDA has called for the inclusion of CASP into clinical studies of blood pressure.^4^ Office CASP could therefore also represent ambulatory CASP well in other CKD-5D populations, although this requires further study. Our observations support findings by other groups where interdialytic measurement of blood pressure was superior to office blood pressure in predicting ambulatory measurements for CKD-5D patients.^31^,^32^

There were several limitations to our study. First, the patients in our cohort were young and one cannot be certain whether these findings would be reproduced in an older cohort. Second, we were not able to measure PWV in our study and it would have been useful to do this in attempting to reconcile the lack of effect of vascular calcification on central aortic pressures. It remains to be determined in this cohort whether vascular calcification occurs independently of changes in pulse-wave velocity. Third, CASP was indirectly measured, although a recent publication showed excellent correlation of BPro with direct measurement of CASP.^17^

## Conclusion

Coronary and abdominal aortic calcification was not associated with changes in central aortic systolic pressure or dipping status in young South African dialysis patients. Inter-dialytic office blood pressure and central aortic systolic pressure, when measured according to ESH standards, correlated very well with ambulatory measurements.
